# Supplementary material for: Charge carrier localised in zero-dimensional (CH3NH3)3Bi2I9 clusters
Source: Nat Commun. 2017 Aug 1;8:170. doi: 10.1038/s41467-017-00261-9 (PMC5537240; doi:10.1038/s41467-017-00261-9)
Supplement: Supplementary file 1 — Supplementary Information [file 41467_2017_261_MOESM1_ESM.pdf]

File Name: Supplementary Information
Description: Supplementary Figures, Supplementary Table, Supplementary Discussion, Supplementary Methods and Supplementary References

File Name: Peer Review File
Description:

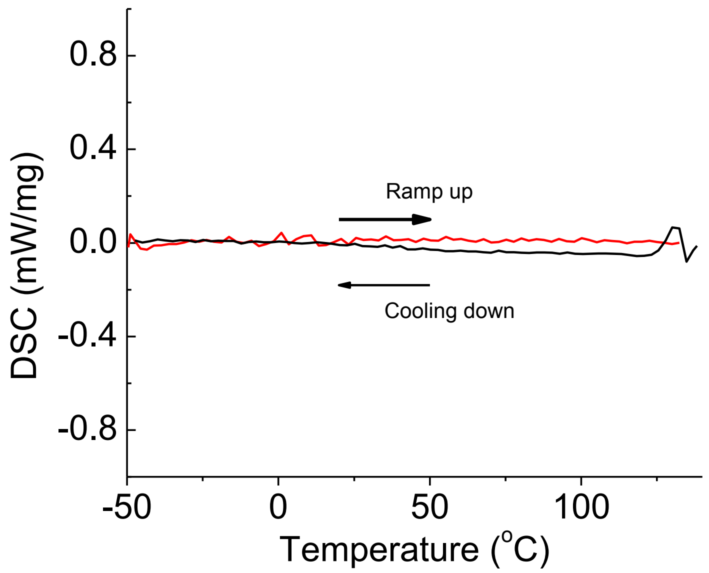

Supplementary Figure 1| Differential Scanning Calorimetry (DSC) DSC measurement of MABI in the temperature range between -50 to 140 °C

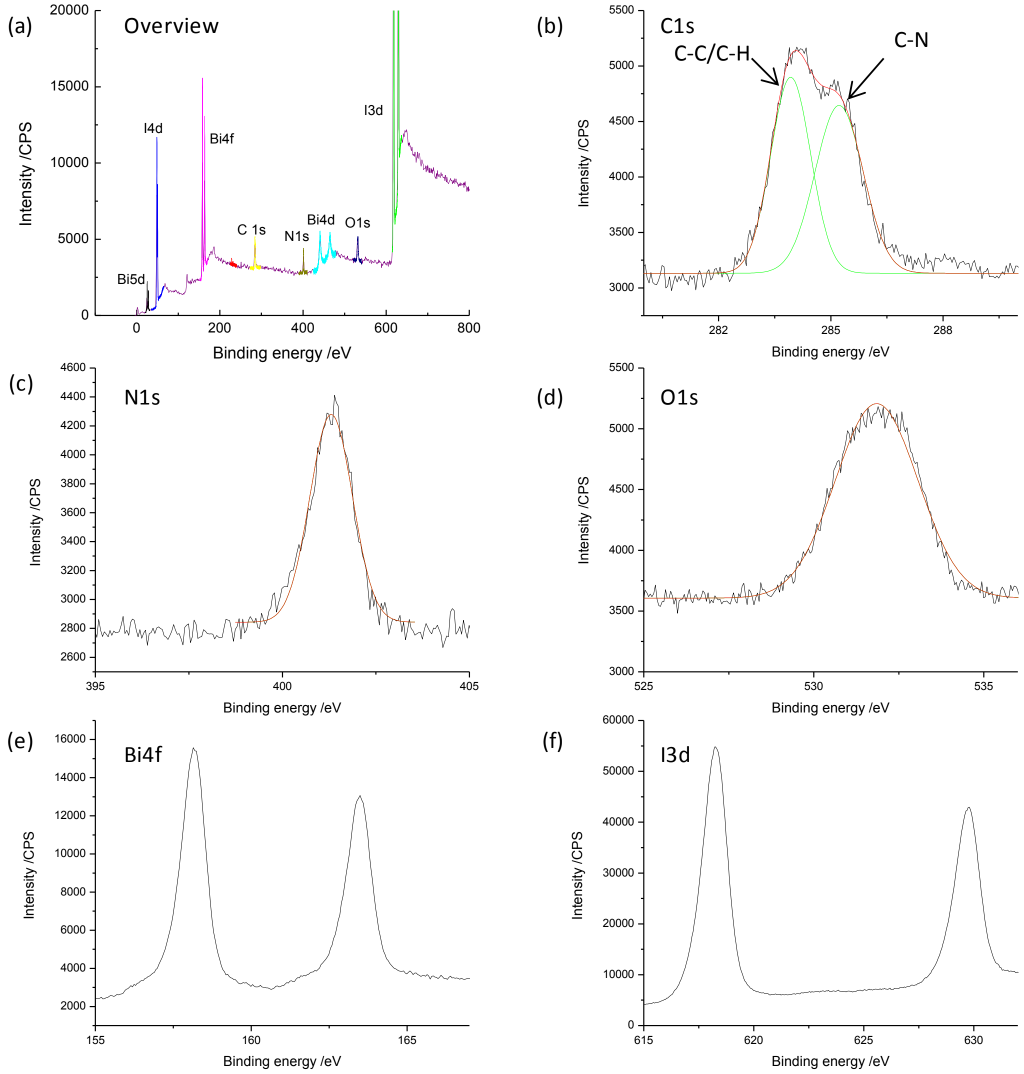

Supplementary Figure 2| X-ray photoelectron spectroscopy (XPS) on MABI coating (**a**) is the overall survey of XPS. (**b, c, d, e, f**) are the respective element scan for C1s, N1s, O1s, Bi4f and I3d. The red lines in (**b, c, d**) show the deconvolution of peaks with Gaussian peak(s).

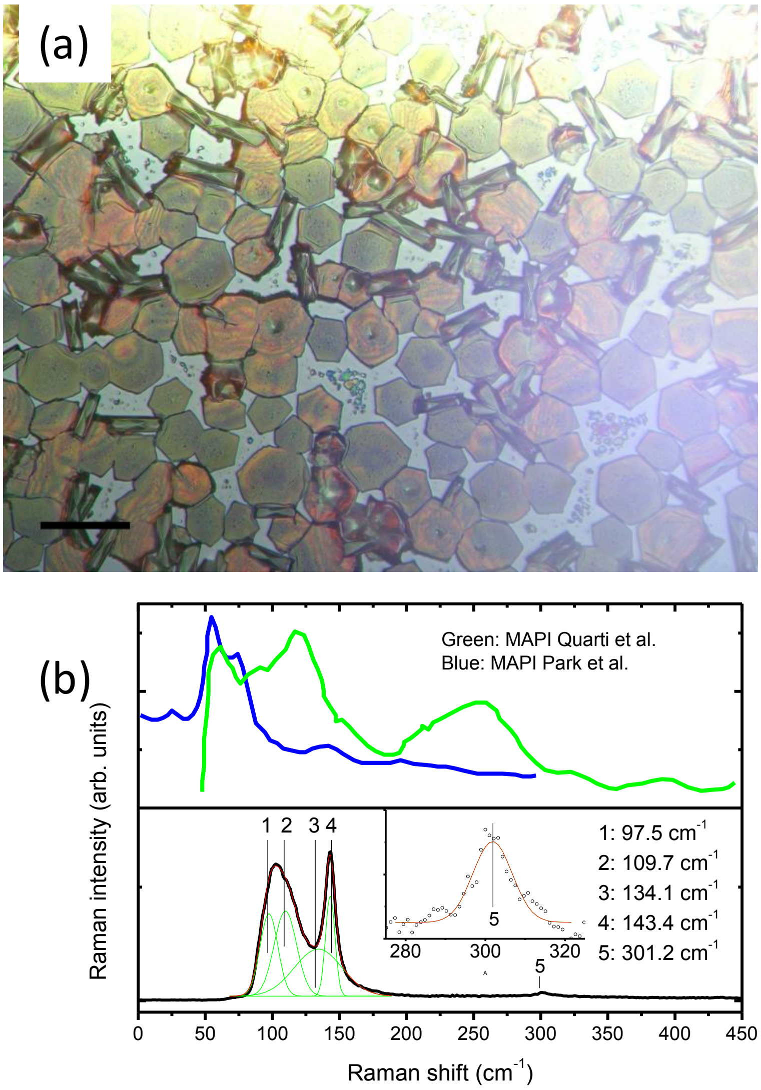

Supplementary Figure 3| Raman spectroscopy of $CH_3NH_3Bi_2I_9$ film (**a**) Optical image of MABI on a silicon single crystal and (**b**) Raman spectra of MAPI (top) from literature and MABI (bottom) in this study. The samples for perovskite were based on MAPI infiltrated in meso-porous alumina (Quarti et al.) or titania (Park et al.).

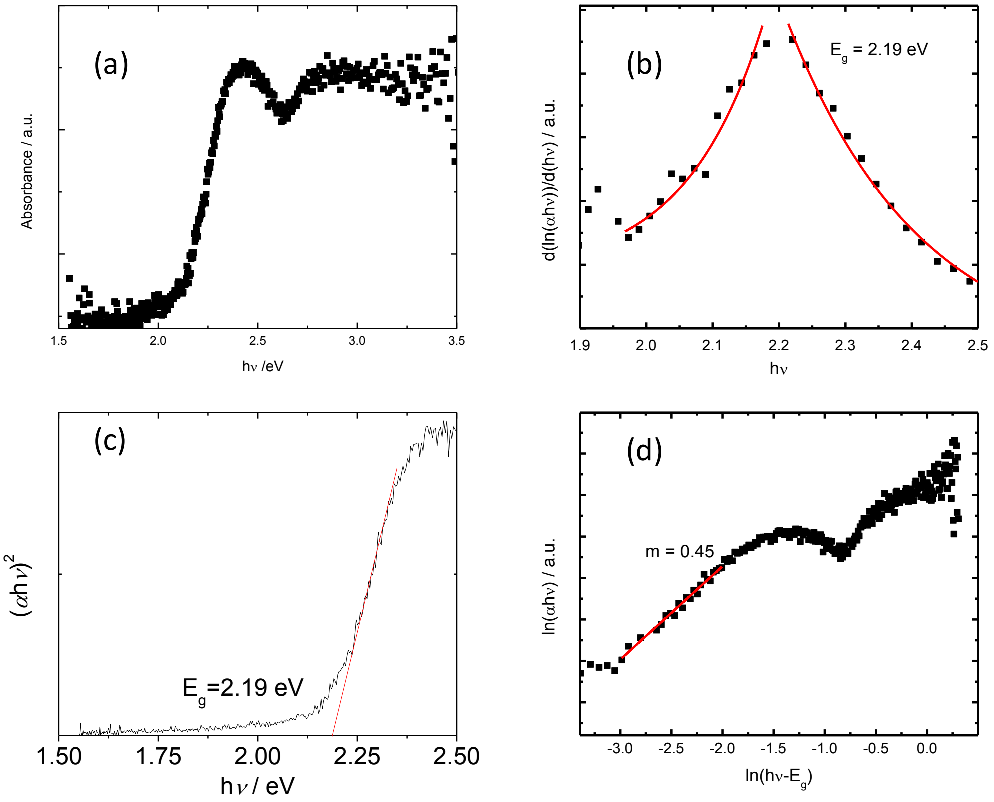

Supplementary Figure 4| Absorbance of coating and determination of band gap (**a**) Absorbance of highly orientated coating converted from transmission using Beer–Lambert law. (**b**) Plot of d(ln(αhν)/d(hν) vs. hν for MABI. (**c**) Tauc plots for the coating for a direct band gap; α is the absorption coefficient. (**d**) Plot of ln (αhν) vs ln(hν-E_g) for E_g = 2.19 eV.

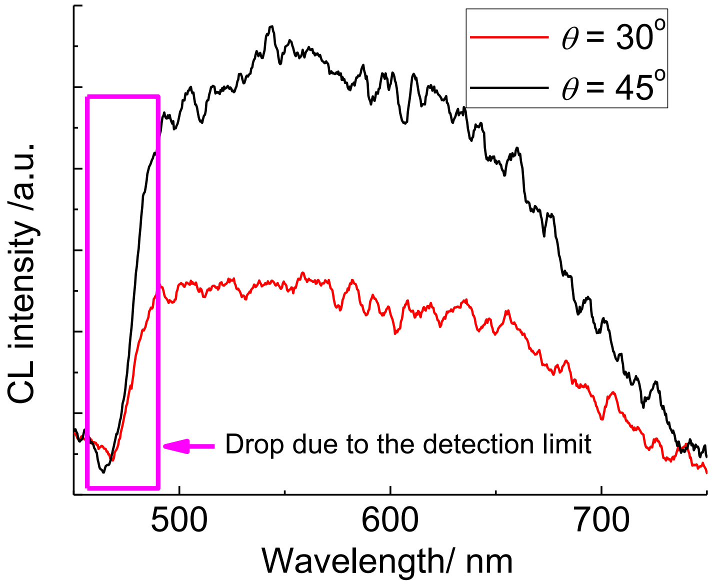

Supplementary Figure 5| Cathodoluminescence of $(CH_3NH_3)_3Bi_2I_9$ (MABI) film Cathodoluminescence of MABI coating under and incident angle of 30° and 45°. The energy of the electron beam is 5 keV, the current is 1.5 nA and the data collection is 30 seconds.

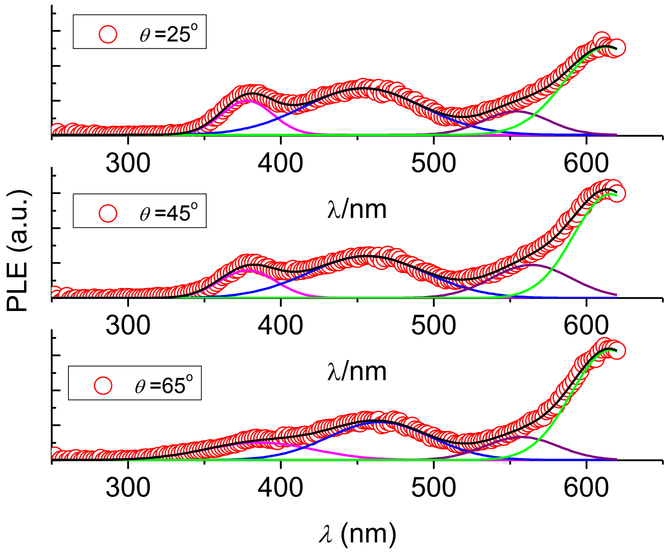

Supplementary Figure 6| Deconvolution of PLE peaks Multiple-peak fitting of PLE spectra at λ_{det} =650 nm at different incident angles.

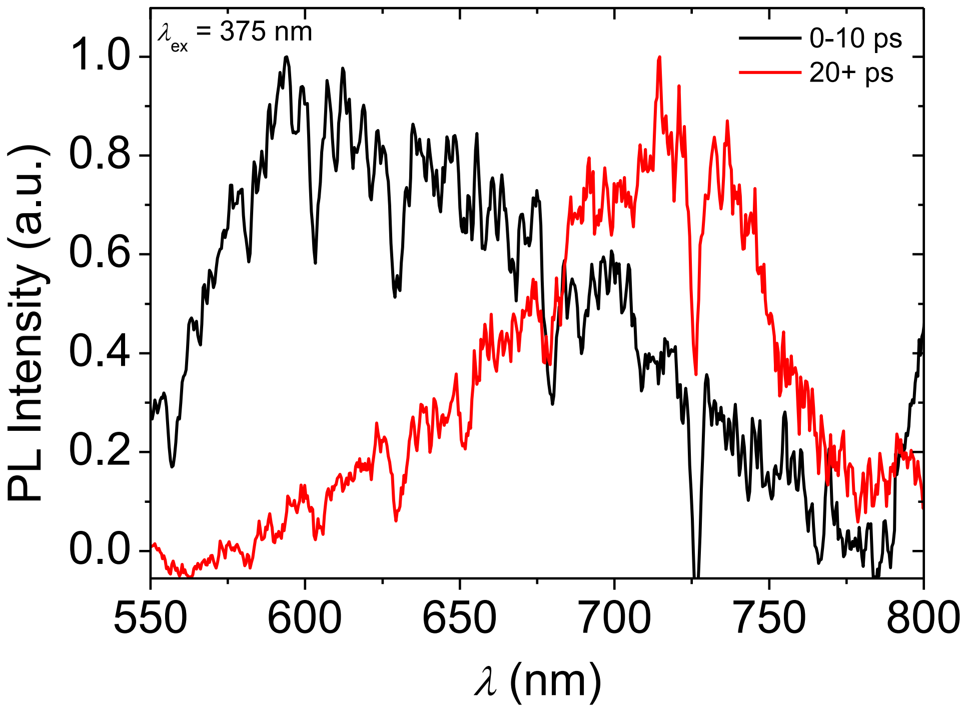

Supplementary Figure 7| Transient Photoluminescence (PL) Transient PL of MABI coating on quartz at λ_{ex} = 375 nm for the excitons with life time between 1 ps and 10 ps and those with life time longer than 20 ps. The spikes are from pump leak.

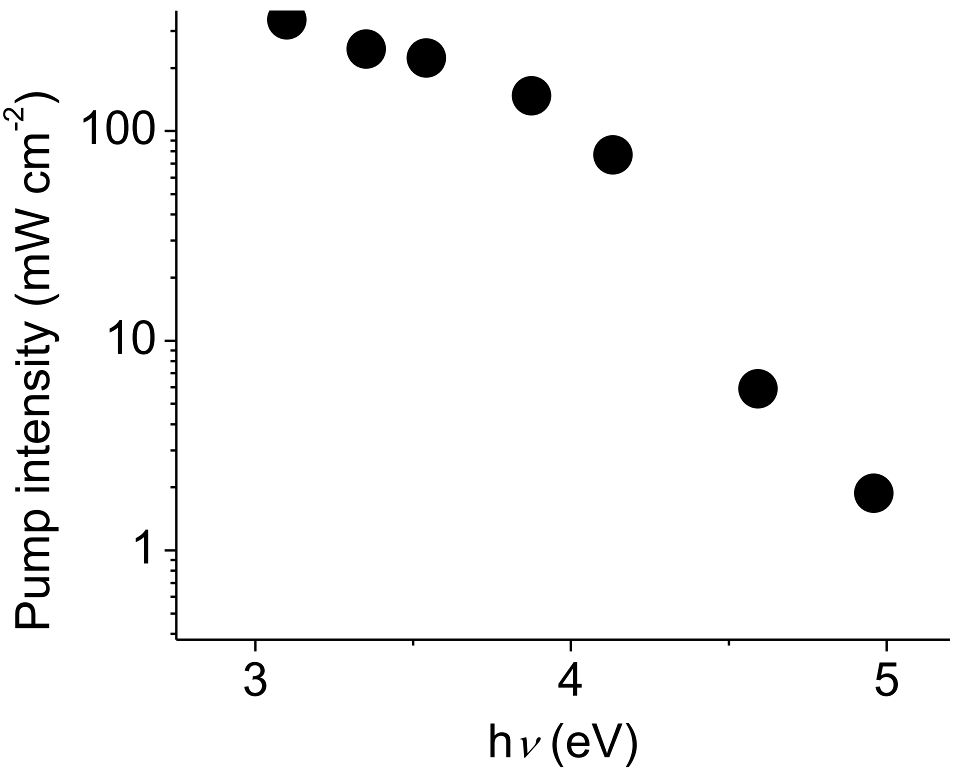

Supplementary Figure 8|. Pump intensities for PL at different excitation energy.

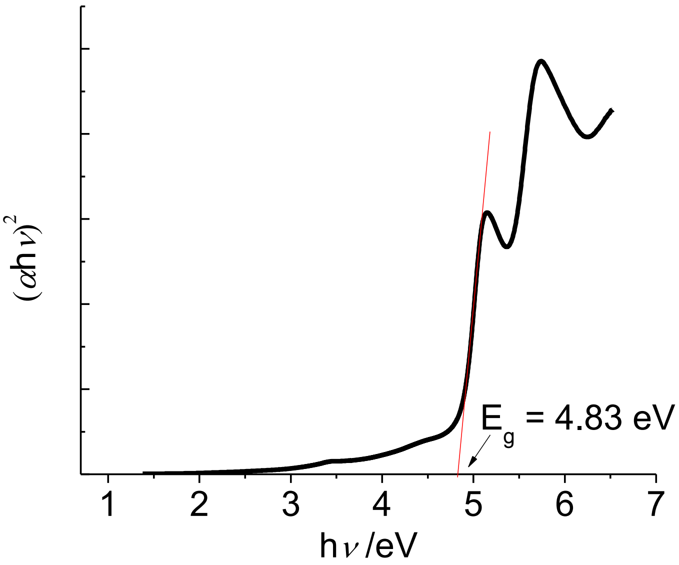

Supplementary Figure 9|. Tauc plots of Methylamine iodide: $(\alpha h\nu)^2$ as a function of photon energy ($h\nu$), where α is the optical absorption coefficient from the diffuse reflectance spectroscopy.

Supplementary Table 1| listing of XPS peaks for the elements of interest.

	Binding energy /eV
C1s	283.92
	285.22
N1s	401.30
O1s	531.85
Bi4$f_{7/2}$	158.27
Bi4$f_{5/2}$	163.51
I3$d_{5/2}$	618.26
I3$d_{3/2}$	629.78

Supplementary Methods

X-ray photoelectron spectroscopy (XPS) XPS measurements were performed using a Kratos Axis Ultra DLD photoelectron spectrometer using monochromatic Al Kα X-ray at an operating pressure of 10^{-9} bar, and current and voltage were 10 mA and 15 kV, respectively. All spectra were acquired at room temperature and were referenced to the Mo3$d_{5/2}$ at 280.0 eV.

Raman spectroscopy Raman spectroscopy of MABI on silicon substrate were performed using a Lab Ram 300 (Horiba Jobin Yvon SAS) and the wavelength of laser was set to be 632.8 nm (HeNe) and the power is tuned to be 0.1 mW to avoid the degradation. Measurement time was 20 seconds for the complete scan for 0-600 cm^{-1}.

Supplementary Discussion

X-ray photoelectron spectroscopy (XPS) The binding energies of XPS peaks were listed in Splintery Table 1. The C1s splits into to peaks and the higher energy one is attributed to C-N bond which is reported to show higher than the C-C/C-H bond.[1] The O1s singlet at 531.85 eV is an indication of absorbed oxygen species rather than that in Bi-O lattice, which shows an O1s binding energy at 530.4 eV.[2] The element ratio of N/Bi/I is calculated to be 3.03/2/8.90 (normalising the composition of Bi to 2), which confirms the right composition of the surface.

Raman spectroscopy of film Raman spectroscopies were performed on MABI on silicon substrate and the microscopy of the crystals was shown in Supplementary Figure 3(a) and the laser is aligned with the centre of hexagonal crystals. The Raman spectrum of MABI (bottom of Supplementary Figure 3(b)) showed distinct feature than MAPI (top of Supplementary Figure 3(b) top). There were fewer peaks in the spectrum for MABI than for MAPI and they can be well fitted into five Gaussian peaks. Four peaks in the region between 75 to 175 cm^{-1} and one at 300 cm^{-1}. In the Raman shift spectrum of MAPI, the peak at 94 cm^{-1} is tentatively assigned to the Pb-I stretching and liberation modes of cations related to inorganic component.[3] The peak at 97.5 cm^{-1} is associated with the Bi-I bridging stretch and it is close to the simulation on $Bi_2I_9^{3-}$ clusters in $(C_6H_{14}N)_3Bi_2I_9$ at 92 cm^{-1} with slight shift to higher energy by around 5 cm^{-1}.[4] The peak at 134.1 cm^{-1} as a broad band is related to the external vibration stretching among the $Bi^2I_9^{3-}$ cluster due to the weak force between the clusters.[4] The sharp peak found at 143.4 cm^{-1} concurring with the Raman shift performed by Park et al was assigned to MA wagging using the DFT calculation based on MA separated PbO_6 octahedra,[5] while Quarti et al. assigned the Raman peak in this region to the motion of organic group in MAPI.[3] Most importantly, the singlet peak at 301.2 cm^{-1} is attributed to the vibrational frequency of the torsional mode of the isolated MA cation at its optimized structure is predicted at 309 cm^{-1} by LDA,[6] which indicates the separation of organic component by the $Bi_2I_9^{3-}$ clusters. The unique singlet in this band can be viewed as a possible marker of the orientational order of the organic cations in the material, and thus of the whole crystal.[3] Interestingly, the peak at energy lower than 75 cm^{-1}, being assigned to the I-Bi-I bending,[4] is not well evolved in our Raman spectra on the crystal planes perpendicular to <002> direction, which could be a result of crystal orientation.

Determination of the band gap of the orientated coating From the absorbance shown in

Supplementary Figure 4(a) we can calculate the bandgap according to references [7, 8]. In the Tauc plot $(\alpha h\nu)^{1/m}$ is plotted against energy (hν), where the nature of the optical transition is represented by the factor m.
With m representing the nature of optical transition in a semiconducting material, the absorption coefficient (α) can be expressed as:

$$\alpha = (A/h\nu)(h\nu - E_g)^m \qquad \text{Equation 1}$$

where A, $h\nu$ and E_g are a constant, the energy of photons and band gap of the semiconductor, respectively.
From equation 1, we then have:

$$d(\ln(\alpha h\nu))/d(h\nu) = m/(h\nu - E_g) \qquad \text{Equation 2}$$

Therefore, if we plot $d(\ln(\alpha h\nu))/d(h\nu)$ vs. $h\nu$, a discontinuity can be observed where $h\nu - E_g = 0$, i.e. at the bandgap value.
Using the data shown in Supplementary Figure 4(a), the differential of $\alpha h\nu$ is taken with respect to $h\nu$ and is plotted against $h\nu$. From this, we can determine the position at which transitions take place, as seen in Supplementary Figure 4(b). Supplementary Figure 4(b) exhibits the discontinuity (that is experimentally shown as a peak) at 2.19 eV, which corresponds to the bandgap transition and is the same as the value from the Tauc plot assuming a direct transition (Supplementary Figure 4(c)). This is already in support of the direct nature of our material.
Furthermore, we can confirm the transition nature by determining the value of the parameter m with the following relation:

$$\ln(\alpha h\nu) = \ln(A) + m\ln(h\nu - E_g) \qquad \text{Equation 3}$$

Taking the gradient of the line gives a value of m = 0.45 which is sufficiently close to 0.5 of a direct transition. We should note that indirect transition would require a value of m =2. Due to the exciton peak, it is not possible to fit the linear portion further, as seen in Supplementary Figure 4(d). Therefore, we have fitted only the linear portion of the curve, which yields a gradient of 0.45.

Supplementary References

1. Rajendra Kumar G*, et al.* Phase transition kinetics and surface binding states of methylammonium lead iodide perovskite. *Phys Chem Chem Phys* **18**, 7284-7292 (2016).

2. Hoye RLZ*, et al.* Methylammonium Bismuth Iodide as a Lead-Free, Stable Hybrid Organic–Inorganic Solar Absorber. *Chemistry – A European Journal* **22**, 2605-2610 (2016).

3. Quarti C*, et al.* The Raman Spectrum of the CH3NH3PbI3 Hybrid Perovskite: Interplay of Theory and Experiment. *The Journal of Physical Chemistry Letters* **5**, 279-284 (2014).

4. Dammak H, Yangui A, Triki S, Abid Y, Feki H. Structural characterization, vibrational, optical properties and DFT investigation of a new luminescent organic–inorganic material: $(C_6H_{14}N)3Bi_2I_9$. *J Lumin* **161**, 214-220 (2015).

5. Park B-w, Jain SM, Zhang X, Hagfeldt A, Boschloo G, Edvinsson T. Resonance Raman and Excitation Energy Dependent Charge Transfer Mechanism in Halide-Substituted Hybrid Perovskite Solar Cells. *ACS Nano* **9**, 2088-2101 (2015).

6. Molecular orbital predictions of the vibrational frequencies of some molecular ions. *The Journal of Chemical Physics* **82**, 333-341 (1985).

7. Chakrabarti S, Ganguli D, Chaudhuri S. Optical properties of γ-Fe2O3 nanoparticles dispersed on sol–gel silica spheres. *Physica E: Low-dimensional Systems and Nanostructures* **24**, 333-342 (2004).

8. Chakrabarti S, Das D, Ganguli D, Chaudhuri S. Tailoring of room temperature excitonic luminescence in sol–gel zinc oxide–silica nanocomposite films. *Thin Solid Films* **441**, 228-237 (2003).
